# Radiopharmaceuticals for Relapsed or Refractory Leukemias

**DOI:** 10.3389/fonc.2019.00097

**Published:** 2019-02-25

**Authors:** Charles A. Kunos, Jacek Capala, Susan Percy Ivy

**Affiliations:** ^1^Cancer Therapy Evaluation Program, National Cancer Institute, Bethesda, MD, United States; ^2^Radiation Research Program, National Cancer Institute, Bethesda, MD, United States

**Keywords:** leukemia, radiotherapy, radiopharmaceutical, radiotherapy - adverse effects, radiotherapy - methods

## Abstract

Radiopharmaceuticals, meaning drugs that hold a radionuclide intended for use in cancer patients for treatment of their disease or for palliation of their disease-related symptoms, have gained new interest for clinical development in adult patients with relapsed or refractory leukemia. About one-third of adult patients outlive their leukemia, with the remainder unable to attain complete remission status following the first phase of treatment due to refractory bone marrow or blood residual microscopic disease. The National Cancer Institute (NCI) Cancer Therapy Evaluation Program conducted 49 phase 1-1b trials in adult patients with leukemia between 1986 and 2017 in an effort to discover tolerated and effective therapeutic drug combinations intended to improve remission and mortality rates. None of these trials involved radiopharmaceuticals. In this article, the NCI perspective on the challenges encountered in and on the future potential of radiopharmaceuticals alone or in combination for adult patients with relapsed or refractory leukemia is discussed. An effort is underway already to build-up the NCI's clinical trial enterprise infrastructure for radiopharmaceutical clinical development.

## Introduction

In 2018, adult leukemias collectively forecast as the eighth most common any-type cancer in Americans (1). Leukemias remain the sixth leading cause of American cancer-related death (1). Leukemias are cancers of the blood and bone marrow, often grouped into three main clusters based on the originating cell type and the pace of cell proliferation—acute myeloid leukemia (AML), chronic myeloid leukemia (CML), and acute lymphocytic leukemia (ALL). The majority (92%) of new leukemia patients arise in adults 20 years of age or older, with one-third of those cases being AML (1). First and subsequent phases of treatment for patients with leukemia are based on a variety of anticancer drug regimens, integrating stem cell transplantation under appropriate conditions. Five-year survival rates for such treated adult patients might be as low as 24 percent for AML and might be as high as 71 percent for ALL (1). For adult AML, the National Cancer Institute (NCI) Cancer Therapy Evaluation Program has launched an aggressive clinical development plan for experimental therapeutic agents (i.e., 49 phase I trials from 1986 to 2017) envisioned to raise overall disease remission and mortality rates. Through new drug discovery, about one-third adult AML patients currently outlive their cancer (2). Those unable to achieve a complete remission after initial treatment, often due to refractory leukemic cells in the bone marrow, die of their disease (2).

A desire to meet therapeutic needs of adult patients with relapsed or refractory leukemias has incentivized one phase of NCI's radiopharmaceutical clinical development plan. The NCI's strategic vision for radiopharmaceutical clinical development considers these agents as radioactive drugs, whereby energy-rich short-range radiation overwhelms a cancer cell's DNA damage response to kill it. NCI puts forward this vision because radiopharmaceuticals have drug-like pharmacology—that is, they demonstrate quantifiable pharmacokinetic exposures and elimination half-lives; they have prescription doses fixed by patient body weight; and they have predictable organ toxicities. By recognizing radiopharmaceuticals as radioactive drugs from the outset, the NCI asserts that they fit better into programmatic concepts around patient safety profiling, efficacy response assessment, and ultimately, disseminating agents for patient care access after completing registration trials.

Innovation, shared commercial partner biomarker co-development, and an opportunity for early phase human trials alone or in unique combination with other drugs within NCI's drug portfolio often piques interest to study patient safety and treatment efficacy early in clinical development. Contrast two approaches. First, the antibody–drug conjugate gemtuzumab ozogamicin (Mylotarg) targeted CD33-positive AML blast cells, was evaluated preclinically (3), obtained accelerated approval (in 2001), but had approval withdrawn (in 2009) when efficacy and toxicity concerns arose (4). Alternatively, the antibody–thorium-227 radionuclide conjugate was crafted to target CD33-positive AML blast cells using the humanized anti-CD33 IgG1 antibody lintuzumab (5–7), underwent preclinical leukemic cell localized and disseminated xenograft mouse modeling and toxicology (8), and is now positioned to enter the clinic for first-in-human pharmacokinetic and pharmacodynamic studies. NCI's radiopharmaceutical clinical development plan envisions antibody–thorium-227 radionuclide conjugate trials that test whether this agent can be safely combined with conventional or experimental therapeutic cytolytic agents.

Therefore, the purpose here is to provide NCI's thoughts to investigators and commercial partners for clinical development of oncology therapeutic radiopharmaceuticals, a term coined elsewhere (9). In this perspective article, NCI first outlays challenges and opportunities encountered in its radiopharmaceutical clinical development venture in AML. Next, a summary of NCI's experience with DNA damage response modifiers prescribed for AML because radiopharmaceutical-agent combination trials might use a rationale of combining two or more well-tolerated agents whose toxicities are considered non-overlapping. NCI's phase I adult leukemia trials of triapine (3-aminopyridine-2-carboxaldehyde thiosemicarbazone or 3-AP), as a potent inhibitor of ribonucleotide reductase (RNR) activity (10, 11), aid discussion of NCI's current thoughts on such combinations. Last, clinical management of adult AML involves repeated blood or bone marrow sampling for assessment of treatment response, and as such, NCI provides guidance on collection and assay of biospecimen samples after patients are given radiopharmaceuticals.

## Challenges and Opportunities

Therapeutic radiation may be administered by treatment beams external to the body, or, by ingested or intravenous formulations of radiopharmaceuticals (hereafter meaning, a radioactive drug product). Radiopharmaceuticals intend targeted delivery of energy-rich radiation (like alpha- [helium nucleus] or beta- [electron] particles) to cancer cells circulating in the blood or in tumors. Targeted delivery means in this case that a ligand, like an antibody or a peptide, binds a radiopharmaceutical onto cancer cells with greater affinity than on normal cells. In the case of alpha-emitting radiopharmaceuticals, the linear energy transfer (LET), or the amount of energy that the ionizing alpha particle transfers to tissue traversed per unit distance, is typically one to ten-cell diameters thick.

NCI's plan to develop clinically targeted radiopharmaceuticals involves establishing new logistics for scientific review, oversight, medical monitoring, and other infrastructure elements fundamental to full agent development. From NCI's perspective, many forward-thinking tactics need consideration prior to starting radiopharmaceutical clinical research. Collaborative research planning among qualified experts in radiation oncology, nuclear medicine, and medical physics; cost sharing strategies with commercial suppliers; radiopharmaceutical drug product formulation and distribution knowledge; and a comprehensive understanding of current treatment recommendations in a desired cancer patient study population; are well-known prerequisites. The NCI is scientifically and academically interested in leading a targeted radiopharmaceutical clinical development program because it provides the opportunity to improve efficient, safe, and cost-effective study in the short and long term. Of note, unique challenges arise in novel radiopharmaceutical development for adult leukemia.

**Figure 1:**
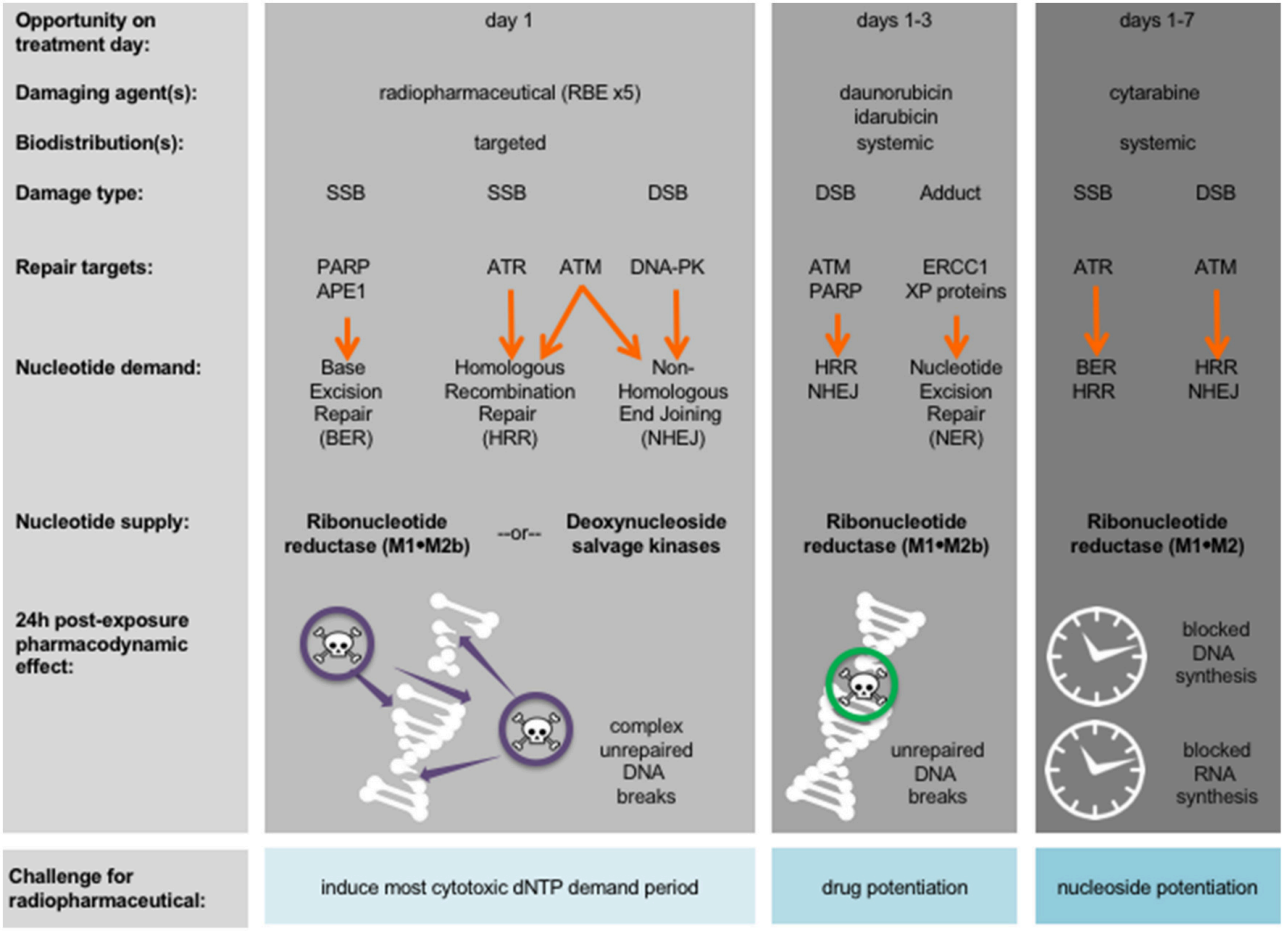
Strategy for radiopharmaceuticals targeting leukemias. Damaging agents and DNA damage response repair targets are charted in relation to proposed radiopharmaceutical-drug agent combinations and treatment days. Shown in bold are nucleotide supply chain elements likely to be active after indicated damaging agent. 24-h post-exposure pharmacodynamic effects after indicated damaging agent are illustrated. APE1, AP endonuclease 1; ATM, ataxia-telangiectasia mutated; ATR, ataxia-telangiectasia and Rad3-related; DNA-PK, DNA-dependent protein kinase; dNTP, deoxynucleotide triphosphate; DSB, double-strand DNA break; ERCC1, DNA excision repair protein 1; PARP, poly(ADP-ribose) polymerase; RBE, relative biologic effectiveness to photon or electron treatment; SSB, single-stand DNA break; XP, xeroderma pigmentosum.

A first aspect of leukemia as a disease that is different from normal cells is that most (if not all) leukemic cells have disrupted nucleotide demand-supply machinery, leading to leukemogenesis (12). This observation suggests then or either a radiopharmaceutical-RNR inhibitor (RNRi) or radiopharmaceutical-nucleoside analog (NA) treatment for leukemia. Figure 1 outlines the underlying disruptions in nucleotide demand and supply as well as the challenges for clinical use of radiopharmaceuticals alone or in combination with modifiers of nucleotide supply for treatment of leukemia. While nucleotide demand-supply chain activation has been proposed as a highly-regulated checkpoint for unrestrained cell growth. In leukemogenesis, this checkpoint abrogated either through a gain in *de novo* RNR output or deoxynucleoside kinase salvage. Before launching into first-in-human radiopharmaceutical combination trials for adults with leukemia, the NCI undertakes scientific review of proof-of-concept studies to show cell affinity and activity. For example, leukemic blasts overexpress RNR (11), deoxynucleoside kinases (13), or the sialoadhesin receptor CD33 (14). Leukemic blasts die *in vitro, ex vivo*, and *in vivo* after exposure to RNR inhibitors (15–18), to nucleoside analogs like cytosine arabinoside (cytarabine) and 9-beta-D-arabinosyl-2-fluoroadenine (fludarabine) activated by deoxycytidine kinase (19, 20), or to an anti-CD33 monoclonal antibody-thorium-227 radionuclide conjugate (8). Additional data considered in the scientific review includes radionuclide pharmacology and cell or organ-seeking properties (like those in Table 1) that may inform the selection of a particular radiopharmaceutical-agent pair for study in an early phase combination trial.

**Table 1:** Pharmacokinetic properties of select radiopharmaceuticals.

**Agent**	**Dose (MBq/kg)**	**Cmax (MBq/kg)**	**Tmax (h)**	**T4h (%)**	**T24h (%)**	**Tp (d)**	**Tb (d)**	**Te (d)**	Mass Dose (μg)^*^	**References**
Radium-223 dichloride	0.055	0.055	< 0.25	4	1	11.4	NA	11.4	NA	(21)
Lutetium-177 dotatate	105.714	105.714	< 0.50	7	0	6.7	NA	6.7	NA	(22)
Anti-CD33 MAb-Thorium-227	0.700	0.700	4	100	83	18.7	7.0	5.1	6.84	(23)

* *Mass dose is the total dose of a non-radioactive or “cold” pharmaceutical, such as the anti-CD33 lintuzumab antibody of the targeted thorium conjugate radiopharmaceutical. Radium and lutetium dotatate are considered neat radionuclides (i.e., contain no ligand)*.

*MBq, megabecquerel; mCi, millicurie; MAb, monoclonal antibody; h, hours; d, days; Cmax, maximum serum concentration; Tmax, time after administration when maximum serum concentration is reached; T4H, proportion of administered dose remaining at 4 h after injection; T24H, proportion of administered dose remaining at 24 h after injection; Tp, physical half-life radionuclide; Tb, biological half-life of ligand; Te, effective half calculated as 1/Tp + 1/Tb = 1/Te; μg, microgram; NA, not applicable*.

A second aspect of leukemia as a disease is the immediacy of starting cytolytic therapy to promote mitotic or apoptotic cell death and to impede leukostasis without undue patient harm (24). Figure 1 identifies periods during conventional leukemia treatment when radiopharmaceuticals, by their ligand affinity or organ-seeking nature, may overcome overlapping drug toxicity. In its programmatic approach to radiopharmaceutical clinical development, the NCI evaluates the frequency of radionuclide decay-related and ligand-related adverse events equally as safety considerations. Preclinical radionuclide biodistribution and ligand toxicology investigations are critical in trial idea “go” or “no-go” decisions. Take as an example an adult patient leukemia trial evaluating a radiopharmaceutical-NA combination. Already common knowledge of radiation-induced organ or marrow toxicities should be considered sufficient to address anticipated radionuclide-related toxicities. Therefore, a radiopharmaceutical that contains no ligand (i.e., a neat radionuclide) should not require preclinical toxicology studies or early phase human trials when it is administered by itself. When a no-ligand radiopharmaceutical is given in combination with other experimental drugs, preclinical toxicology studies, and early phase human trials are justified. Beyond just the radionuclide, radiopharmaceutical ligand-related toxicology should be considered. “Cold” non-radioactive pharmaceutical toxicology studies should document any clinical manifestations, altered body weight, or changes in hematological or serum chemistry markers.

Tables 2, 3 tie together toxicities encountered in NCI's triapine-fludarabine (17) and triapine-cytarabine (18) trials, and, the ligand-related toxicities of lintuzumab (23), an anti-CD33 monoclonal antibody that is part of a targeted thorium-227 conjugate (8), in the context of no-ligand radium or lutetium. The first NCI phase 1b trial tested the sequence of *de novo* RNRi by triapine followed by NA exposure by fludarabine (17). Twenty-four eligible adult patients with relapsed refractory leukemia were allocated to 5-day triapine (105 mg m^−2^) then immediate dose-escalated fludarabine (15–30 mg m^−2^), recurring every 21 days. The leukemia complete remission rate was eight percent (2 of 24). The second NCI phase 1b trial enrolled 25 adult patients with relapsed or refractory leukemia for treatment by cytarabine (1,000 mg m^−2^) on days 1–5 in combination with dose-escalated triapine (50–100 mg m^−2^) on days 2–5, repeated on a 28-day cycle (18). The complete remission rate of leukemia was eight percent (2 of 25). Both trials offered the opportunity to learn what toxicities might be encountered in a future radiopharmaceutical-RNRi or NA combination trial. This opportunity is now discussed based on three common risk-based toxicities.

**Table 2:** Drug-related common adverse events in the study population^*^.

**Event**	**Triapine**		**Triapine-Fludarabine**		**Triapine-Cytarabine**	
	**(*****N*** **=** **49)**		**(*****N*** **=** **24)**		**(*****N*** **=** **25)**	
	**Any**	**Grade**	**Any**	**Grade**	**Any**	**Grade**
		**3 or 4**		**3 or 4**		**3 or 4**
Any events	49 (100)	25 (51)	21 (88)	11 (46)	21 (84)	16 (64)
Neutropenia/sepsis	5 (10)	5 (10)	12 (50)	8 (33)	12 (48)	12 (48)
Leukopenia	21 (43)	17 (35)	12 (50)	9 (38)	20 (80)	20 (80)
Thrombocytopenia	6 (12)	3 (6)	9 (38)	7 (29)	17 (68)	17 (68)
Anemia	9 (18)	3 (6)	16 (67)	7 (29)	16 (64)	16 (64)
Fatigue or asthenia	17 (35)	0	10 (42)	0	8 (32)	1 (4)
Nausea	31 (63)	1 (2)	18 (75)	0	17 (68)	0
Diarrhea	13 (26)	1 (2)	9 (38)	1 (4)	9 (36)	0
Constipation	12 (24)	0	0	0	1 (4)	0
Vomiting	29 (59)	2 (4)	15 (63)	1 (4)	7 (28)	0
Pyrexia	38 (78)	2 (4)	12 (50)	1 (4)	5 (20)	0
Pain	12 (24)	0	2 (8)	0	1 (4)	0
Rigor/flushing	15 (31)	0	5 (21)	0	7 (28)	1 (4)
Increased ALT/AST	25 (51)	6 (12)	10 (42)	1 (4)	0	0
Headache	13 (27)	1	0	0	0	0
Cough	11 (22)	0	1 (4)	0	0	0
Methemoglobinemia	1 (2)	0	1 (4)	0	4 (16)	0
Reference	(25, 26)		(17)		(18)	

* *Patients could have more than one adverse event. The safety population included all patients who received at least one dose of a study drug*.

**Table 3:** Common adverse events in the study population^*^.

**Event**	^**223**^**Radium**		^**177**^**Lutetium**		**Anti-CD33 MAb-**^**227**^**Thorium**	
	**(*****N*** **=** **600)**		**(*****N*** **=** **111)**		**(*****N*** **=** **23)**	
	**Any**	**Grade**	**Any**	**Grade**	**Any**	**Grade**
		**3 or 4**		**3 or 4**		**3 or 4**
Any events	558 (93)	339 (56)	105 (98)	46 (41)	18 (78)	2 (20)
Neutropenia/sepsis	30 (5)	13 (3)	6 (5)	1 (1)	1 (4)	1 (4)
Leukopenia	NR	NR	4 (10)	1 (1)	0	0
Thrombocytopenia	69 (12)	38 (5)	28 (25)	2 (2)	0	0
Anemia	187 (31)	76 (13)	16 (14)	0	0	0
Fatigue or asthenia	154 (26)	24 (5)	44 (40)	2 (2)	0	0
Nausea	213 (36)	10 (2)	65 (59)	4 (4)	5 (22)	0
Diarrhea	151 (25)	9 (2)	32 (28)	3 (3)	0	0
Constipation	108 (15)	6 (1)	14 (13)	0	0	0
Vomiting	111 (18)	10 (2)	52 (47)	8 (7)	6 (26)	0
Pyrexia	38 (6)	3 (1)	NR	NR	12 (52)	0
Pain	300 (50)	125 (21)	32 (29)	2 (2)	5 (22)	1 (4)
Rigor/flushing	NR	NR	14 (13)	1 (1)	7 (30)	0
Increased ALT/AST	NR	NR	0	0	3 (13)	0
Headache	NR	NR	18 (16)	0	3 (13)	0
Cough	NR	NR	12 (11)	0	1 (4)	0
Methemoglobinemia	NR	NR	NR	NR	NR	NR
Reference	(27)		(28)		(5, 6)	

* *Patients could have more than one adverse event. The safety population included all patients who received at least one dose of a study drug. Common adverse events for the anti-CD33 MAb-^227^Thorium conjugate are listed for the antibody ligand alone, as the conjugate is just beginning early trial phase clinical development*.

*MAb, monoclonal antibody; NR, not reported*.

Anemia may arise from either greater destruction or diminished production of red blood cells. It manifests as easy fatigue, and in severe cases, can lead to dyspnea, tachycardia, and headache. Of all patients treated on the NCI's triapine-NA trials, 67 percent developed anemia (17, 18). In contrast, the bone-seeking radiopharmaceutical radium dichloride (simply, radium, or ^223^Ra) led to anemia in only 31 percent of patients (27) while the somatostatin receptor positive tumor-seeking lutetium dotatate (lutetium or ^177^Lu) resulted in only a 14 percent rate of anemia (28). Supplements or blood transfusions may be indicated if blood production is adversely affected.

Fatigue or exhaustion might result from skeletal muscle expending energy to furnish nucleosides via the bloodstream to cells requiring increased nucleoside supply (29). About one-third of all patients treated on NCI's triapine-NA trials had fatigue (17, 18). Any-grade fatigue after radium (26%) or lutetium (40%) was less frequent (27, 28). The NCI is particularly interested in fatigue as a patient-reported outcome (30), and, it might possibly be listed as a special interest dose-limiting toxicity in its future radiopharmaceutical trials, because of the complex relationship of nucleoside demand-supply responses among normal cells and skeletal muscle is presumably unknown. By anecdote, high protein diets and light exercise boost energy in such situations. Further study is warranted.

Nausea, vomiting, diarrhea, and constipation are all common toxicities encountered after therapies for adult leukemia. In the NCI's triapine-fludarabine trial, patients reported 44 percent nausea, 36 percent vomiting, 22 percent diarrhea, and zero percent constipation of any grade (17). In the NCI's triapine-cytarabine trial, patients reported 68 percent nausea, 44 percent vomiting, 36 percent diarrhea, and four percent constipation of any grade (18). Of note, radium can be excreted by luminal cells of the intestine and thus might accumulate in stool. Radium associates with rates of 36 percent nausea, 18 percent vomiting, 25 percent diarrhea, and 18 percent constipation (27), which is lower than expected. Lutetium can track to normal endocrine cells and nerve fibers of the gastrointestinal tract. It thus has higher frequencies of nausea (59%), vomiting (47%), diarrhea (29%), and bowel distension (13%) (28). Based on these observations, NCI considers gastrointestinal adverse events of special interest that will require additional toxicity monitoring and reporting, especially for radiopharmaceuticals targeting or eliminated by the intestines. Opioid-related constipation is another clinical situation that requires monitoring and reporting in the NCI's perspective for radiopharmaceutical clinical development.

## Perspectives on Radiopharmaceutical Biomarker Development

**Figure 2:**
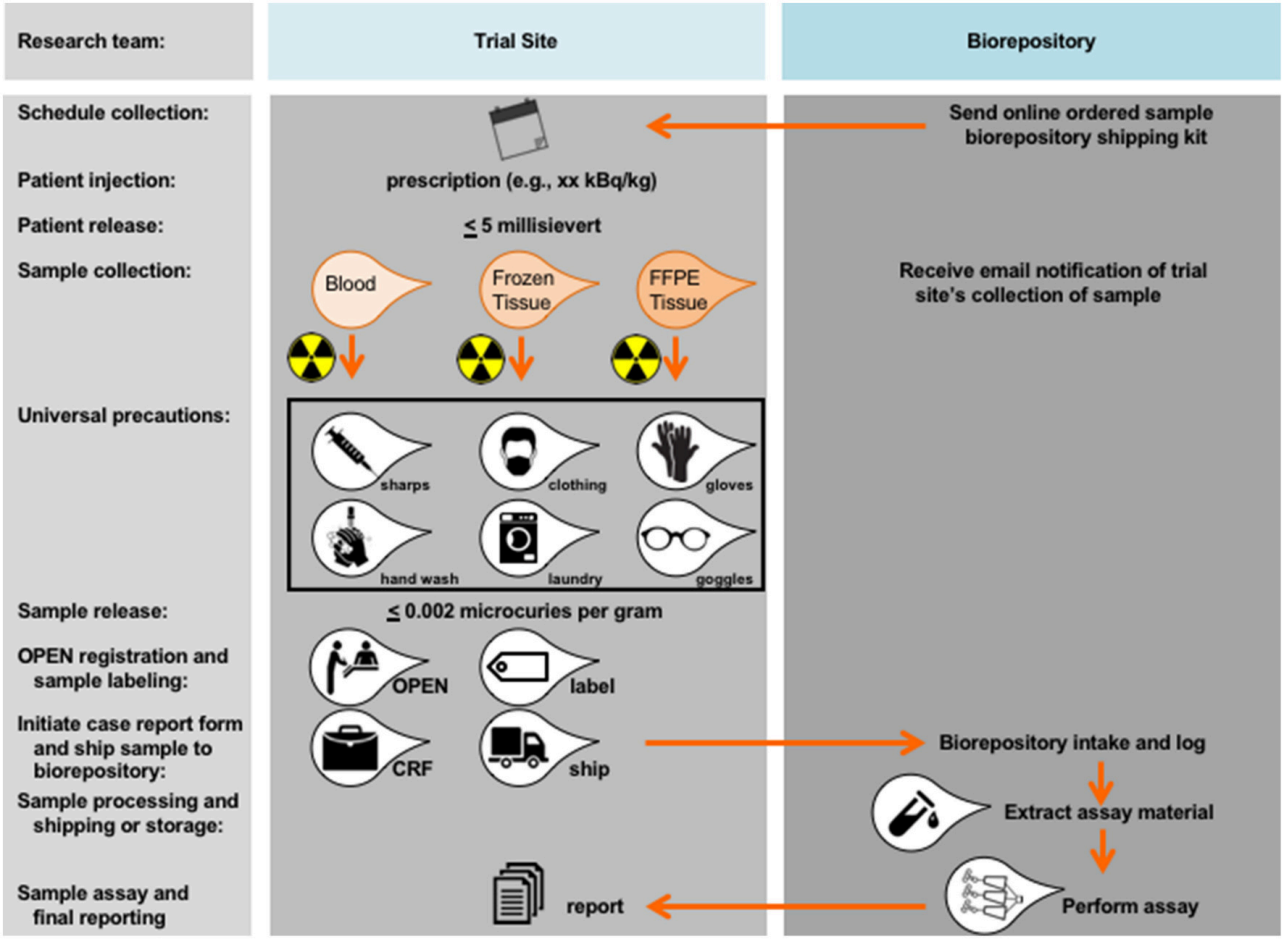
Proposed workflow for blood and tissue biomarker development in radiopharmaceutical trials. Proposed workflow steps are charted in relation to the trial site or the biorepository. As a first step, blood or tissue collection should be scheduled as indicated and relative to date of planned radiopharmaceutical administration. Shipping kits (if any) should be acquired by the trial site from the biorepository. A trial site licensee may authorize the release from its control any individual who has been administered an unsealed radiopharmaceutical when the total effective radiation dose equivalent to any other individual from exposure to the released individual is unlikely to exceed 5 millisievert (0.5 rem). There are no guidances that preclude a blood draw or tissue sampling after an individual has been released under these stipulations. Universal precautions should be used, meaning any team member handling a sample should be wearing non-porous medical gloves, goggles, and face shields, should wash hands using soap under a steady stream of water for at least 10 s, should launder soiled clothing or linens (that are handled with gloved hands), and should dispense of needles or sharp instruments in puncture-resistant containers designed for such purposes.

The NCI has fast tracked organizational builds around radiopharmaceutical scientific review and biomarker development. One need is qualified expert appraisal by radiation oncology or nuclear medicine physicians at initial NCI Experimental Therapeutics (NExT) program applicant scientific review. By leveraging programmatic collaboration, the NCI should render early “go” or “no-go” decisions without extensive resource provision. A second need is a method for blood or tissue acquisition and processing after radiopharmaceutical administration (Figure 2). NCI played out scenarios to think through the consequences of blood or tissue sampling in a radiopharmaceutical-experimental drug trial. The NCI often uses its biorepository partner (like the Experimental Therapeutics Clinical Trials Network [ETCTN] biorepository) and laboratory partners (like the Frederick National Laboratories-Molecular Characterization Laboratory [https://frederick.cancer.gov/science/clinical] or Pharmacodynamic Assay Development/Implementation Section [https://next.cancer.gov/developmentresources/pd_biomarker.htm]) to support these tasks. For guidelines on handling blood and tissue samples after radiopharmaceutical administration, the NCI already has radiation safety officer and U.S. Nuclear Regulatory Commission procedures in place. From its perspective, patients administered radiopharmaceuticals are fully releasable when conditions specified under 10 CFR 35.75 are met, which means that a radioactive licensee (meaning, the administering physician) may authorize the release from its control any individual who has been administered an unsealed radiopharmaceutical when the total effective radiation dose equivalent to any other individual from exposure to the released individual is unlikely to exceed five millisieverts (5 mSv or 0.5 rem). Assuming an individual who received a radiopharmaceutical has been discharged from the administering physician, the NCI finds no current guidances that preclude post-therapy blood draw or tissue biopsy if these stipulations are met. As with any instance of blood or tissue collection (radioactive or non-radioactive), universal precautions should be used. Universal precautions pertinent to radiopharmaceutical administration means (a) the practice of avoiding contact with a patients' blood or tissue by wearing non-porous medical gloves, goggles, and face shields, (b) hand washing using soap and a steady stream of water for at least 10 s, (c) laundering soiled clothing or linens (handled with gloved hands), and (d) dispensing needles or sharp instruments in puncture-resistant containers (Figure 2). Personnel potentially collecting radioactive blood (like in trials rapidly drawing blood samples for pharmacokinetics) should have basic radiation safety training commensurate with their potential radiation exposure. Biospecimens are fully releasable for shipment without radioactive labeling when conditions under 48 CFR 52.223–7 are met (≤ 0.002 microcuries per gram, www.govinfo.gov).

## Conclusion

In summary, this perspective article examines the scope of radiopharmaceutical clinical development as related to use in adult leukemias; the evaluation of radionuclide and ligand toxicities as related to targeted radiopharmaceuticals; and guidance on the acquisition, handling, processing, and shipment of biomarker samples after radiopharmaceutical administration.

The current NCI position does not address radiopharmaceuticals that might be considered for leukemia treatment that are intended for a local route of administration (e.g., intratumoral or intraosseous marrow routes of administration). Important overarching topics related to radiopharmaceutical drug product specification or impurity, stability, handling and distribution, or multistep labeling kits (e.g., making an antibody-radiopharmaceutical conjugate immediately before human use) are not discussed here. Guidances for such topics are found elsewhere (9). The NCI view on radiopharmaceuticals considers both radionuclide and ligand toxicities, especially as adverse events relate to the biodistribution of emitted energy-rich radiation in targeted and in non-targeted organs. Educating patients and clinical providers about radiopharmaceuticals remains necessary for beneficial clinical development in adult leukemia.

## Ethics Statement

The research presented in this article involved the collection or study of existing data, documents, and records that were publicly available, or the information was recorded by NCI in such a manner that trial subjects cannot be identified directly or through identifiers linked to the subjects. The research is regarded exempt from Institutional Review Board oversight.

## Author Contributions

CK, JC, and SI contributed to the collection and review of any perspective or trial data, analysis, and authentication, and the writing and approval of this manuscript. The views expressed are those of the authors and not those of the U.S. federal government. Links or discussion of specific radiopharmaceutical drug products do not constitute endorsement.

### Conflict of Interest Statement

The authors declare that the research was conducted in the absence of any commercial or financial relationships that could be construed as a potential conflict of interest.
